# Occipital nerve stimulation in pediatric patients with refractory occipital neuralgia

**DOI:** 10.1007/s00381-024-06376-x

**Published:** 2024-04-02

**Authors:** James Mossner, Nour B. Saleh, Maryam N. Shahin, Joshua M. Rosenow, Jeffrey S. Raskin

**Affiliations:** 1 Department of Neurological Surgery, Northwestern University Feinberg School of Medicine, Chicago, IL USA; 2 School of Medicine, New Giza University, Giza, Egypt; 3 Department of Neurological Surgery, Oregon Health & Science University, Portland, OR USA; 4 Division of Pediatric Neurosurgery, Department of Neurological Surgery, Ann & Robert H. Lurie Children’s Hospital, Northwestern University Feinberg School of Medicine, 225 E Chicago Ave, Chicago, IL 60611 USA

**Keywords:** Occipital nerve stimulation, Occipital neuralgia, Pediatric, Pain, Adolescent

## Abstract

**Purpose:**

Occipital neuralgia (ON) is a disabling problem within the pediatric population. Many of these patients fail medical therapies and continue to suffer without further surgical management. Occipital nerve stimulation (ONS) is used to treat ON in the adult population leading to a 72–89% reduction in pain; however, there are limited studies regarding its use in the pediatric population. In this study, we examined the outcomes of ONS in pediatric patients with medically refractory ON.

**Methods:**

We performed a chart review of pediatric patients at our institution who have undergone ONS for the same indications.

**Results:**

We identified 3 patients at our institution who underwent ONS trial and/or permanent implantation for ON. One patient had complete pain relief after the trial and declined permanent implantation. The other patient had fewer attacks compared to his pre-trial baseline and controlled them by adjusting his permanent implant stimulation settings. The last patient had near complete relief of her symptoms and no longer required any pain medication.

**Conclusion:**

Our study highlights the paucity of studies evaluating the utility of ONS in the pediatric ON population. Limited data from both the literature and our institution’s experience reveal that pediatric patients may benefit from trial and/or permanent implantation of ONS for medically refractory ON pain.

**Supplementary Information:**

The online version contains supplementary material available at 10.1007/s00381-024-06376-x.

## Introduction

Chronic occipital neuropathic pain is a pervasive and disabling problem within the pediatric population with a USA population-based estimate of 17% prevalence of all debilitating headaches [1–4]. Occipital pain is considered a “red flag” symptom, prompting clinicians to rule out etiologies requiring emergent interventions [4]. The more common and nonemergent etiologies include pain secondary to occipital neuralgia, Chiari malformation, paroxysmal hemicrania, chronic migraines, and trauma. First-line treatment consists of conservative and pharmacological therapies followed by occipital and other nerve blocks, as well as botulinum toxin injections for refractory cases [5–7]. Unfortunately, many patients who undergo anesthetic nerve blocks or steroid injections will have recurrence of their symptoms despite repeated treatments [8]. In these refractory patients, surgical interventions can be employed. Occipital nerve stimulation (ONS) is used in the adult population as a minimally invasive neuromodulatory therapy for chronic cranial neuropathic pain, occipital neuralgia, migraine, post-herpetic neuralgia, and cervicogenic headaches [9–12]. Studies of ONS have demonstrated a 72–89% reduction in pain in subjects compared to their pre-operative baseline; however, there is a paucity of studies regarding the safety and efficacy of ONS in the pediatric population [10, 13–16]. In fact, in this study, we performed systematic literature review and could not identify a single article dedicated to the use of ONS in pediatric ON patients. Therefore, in this retrospective chart review, we examined our outcomes of occipital nerve stimulation in patients with medically refractory occipital neuralgia.

## Methods

### Search strategy and study selection

We conducted a comprehensive literature search through four large, widely accessed databases (PubMed, Cochrane, Web of Science, and SCOPUS) using the Preferred Reporting Items for Systematic Review and Meta-Analyses (PRISMA) statement in August 2023 [17]. Search terms included “implantable neurostimulators,” “peripheral nerve stimat,” occipital nerve stimulat,” and electric stimulation therapy,” as well as terms that captured a pediatric population, including “adolescent,” “pediatric,” or children.” In addition, our search included the occipital neuralgia diagnosis as “occipital neuralgia.” Our complete search algorithm can be accessed in Supplementary Fig. 1. One author (J.M.) screened for titles, abstracts, and full texts of the resulting articles.

### Institutional chart review

We reviewed demographic, surgery, and outcome data of occipital nerve stimulator placement used to treat three patients with pediatric occipital headaches between 2022 and 2023 (Table 1). This study was approved by the local institutional review board with a waiver of consent. All statistical analyses were performed in RStudio (RStudio Team, 2023).

**Table 1:** Summary of pediatric occipital nerve simulation cases and outcomes

**Patient, age, sex**	**Diagnosis**	**Previously attempted therapies**	**Baseline pain description**	**Unilateral vs bilateral electrode trials**	**Post-trial pain score**	**Outcome**	**Complications**
15 yr, M	Post-traumatic occipital neuralgia	Sumatriptan, nerve blocks, Botox injections	VAS 10/10; attacks 4 days/week; prevents patient from attending school	Bilateral	VAS 1/10	Underwent permanent bilateral electrode implantation; now has 2 headaches/week relieved with stimulation	None
15 yr, F	Idiopathic occipital neuralgia	Propranolol, naratriptan, Botox injections, Toradol injections, nerve blocks, electrolyte infusions, pressure point ear piercings	VAS 10/10; attacks 3–4 days/week; prevents patient from attending school	Bilateral	VAS 0/10	Pain free following trial; elected to not undergo implantation; pain medications reduced by 50% after trial	None
17 yr, F	Idiopathic occipital neuralgia	Duloxetine, ibuprofen, meloxicam, CBD, TENS unit, gabapentin, Lyrica, magnesium, tramadol, nerve blocks	VAS 9/10; requires around-the-clock pain medication	Bilateral	VAS 1/10	Underwent permanent bilateral electrode implantation; no longer takes any pain medication	None

### Surgical technique

#### Trial implant

Following a full informed consent, patients are brought to the operating room and placed prone, awake. We trace the greater and lesser occipital arteries as surrogate markers of the occipital nerves using ultrasonography. Two trajectories targeting the mastoidal tip and the superior nuchal line traversing the course of the arteries are planned from midline. Following a 4-step skin preparation, sterile draping, and surgical pause, we anesthetize the entry points with 0.5% lidocaine and then introduce curved 14-guage Tuohy needles into the subcutaneous and epifascial space. Fluoroscopy confirms placement. We remove the stylet and place 8-contact cylindrical electrodes to the depth of the needle tip and remove the needle. The electrodes are connected to the external neurostimulator which is turned on with escalating current to confirm physiologic coverage of the typical pain pattern. Following confirmation of paresthesia coverage, fluoroscopy is used to record the final tip location and the electrode is sutured down to the skin with a 2–0 nylon suture at both the exit site and a second winged silastic anchor site. We further secure the electrodes with Tegaderm™ and secure them with a dressing. The trial lasts 7 days; at the trial end, the electrodes are removed in clinic and steri-strips are placed over the electrode exit site(s).

#### Permanent implant

The patient is positioned the same as in the trial implantation, this time under general anesthesia. The 4-step skin preparation now includes the back and flank. Landmarks are identified as in the trial. A midline incision allows surgical access to the nuchal fascia, and the Tuohy needles are positioned exactly as in the trial. The electrodes are placed through the needles, which are then withdrawn, using fluoroscopy to ensure stable position of the electrodes. An anchoring system is placed around the leads, and they are secured to the fascia with 2–0 Ti-cron stitches. The flank incision is opened sharply, a subcutaneous pocket is created, and the electrodes are tunneled between the incisions. The electrodes are connected to the pulse generator (IPG), the impedances are checked, and then the electrode and IPG are placed into the pocked and secured. A final fluoroscopy picture confirms placement compared to the trial, followed by wound irrigation with vancomycin irrigation then placement of vancomycin antibiotic powder. We change our gloves and close with antimicrobial suture in layered fashion.

## Results

### Systematic review

**Fig. 1:**
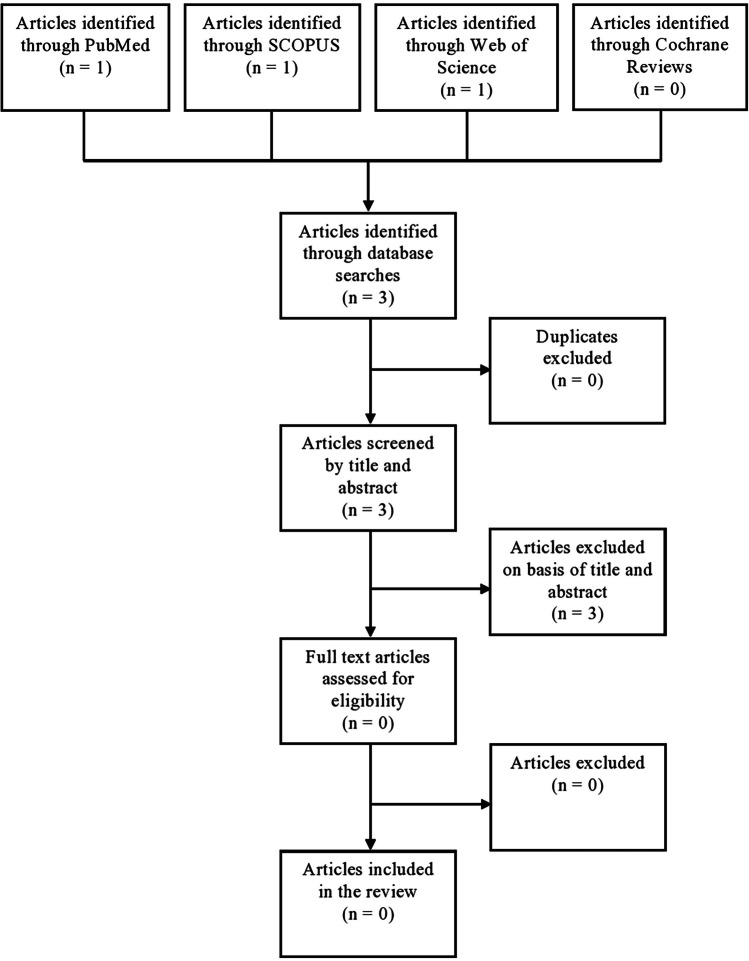
PRISMA flowchart for the literature review

A total of 3 articles (1 from PubMed, 1 from SCOPUS, 1 from Web of Science, and 0 from Cochrane Reviews) were captured by our literature search (Fig. 1), of which none of the 3 articles were specifically dedicated to outcomes of ONS in pediatric ON patients.

### Patient outcomes

**Fig. 2:**
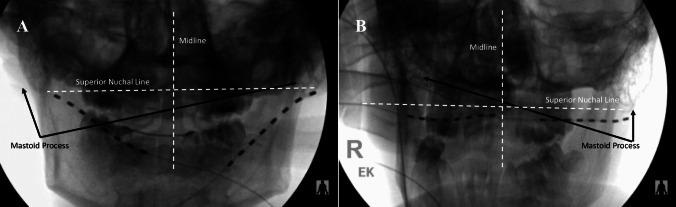
Occipital nerve trial lead placements: intraoperative fluoroscopic images of the patients’ percutaneous trial leads (**A**, **B**)

Our chart review yielded a total of 3 patients (2 female) with an average age of 15.6 years old (range of 15–17 years old). One of these patients had a diagnosis of post-traumatic ON secondary to a football accident. The remaining two patients had a diagnosis of idiopathic ON. All 3 patients rated their pain on the VAS between 9 and 10/10 that had a severe impact on their daily quality of life. In fact, two of the three patients were unable to attend school because of the pain. The frequency of their pain episodes ranged from having attacks 4 days/week to 3–4 attacks/day. All patients had attempted conservative therapies including medications and nerve blocks. All 3 patients had initial symptom relief with anesthetic nerve blocks but, unfortunately, became refractory over time. Each patient underwent a bilateral, percutaneous ONS trial and had remarkable success with a significant reduction in post-trial VAS scores (mean post-VAS 0.67, *p* = 0.002, paired Student’s *T*-test). One patient remained pain-free following her trial and did not undergo permanent implantation. This phenomenon is not understood, and she remains pain-free at 15 months post-operatively. The other two patients, however, did elect to undergo permanent implantation. One of the patient’s attacks persisted following permanent implantation but they were less intense in severity, occurred less frequently, and were able to be aborted with stimulation. The other patient had a 95% reduction in her pain following permanent implantation and no longer required the use of any pain medications to control her symptoms at 9-month follow-up. There were no complications with any of the trial or permanent implantation procedures (Fig. 2).

## Discussion

Our study reviews the indications for and outcomes of ONS in pediatric patients with medically refractory ON. A literature review could not identify a single article pertaining to outcomes of ONS in pediatric ON patients. However, our retrospective chart review identified 3 patients who had a significant reduction in both their pain and pain medication usage following ONS trial and/or permanent implantation.

The neurosurgical management for medically refractory ON in adults includes both ablative and neuromodulatory techniques; recent reviews for different approaches to treating ON and similar conditions in adults highlight both invasive and non-invasive treatment modalities [5, 18]. Open surgical neurectomy and C2 ganglionectomy have long been performed with some benefit; the natural evolution of open-to-minimal-access techniques has brought radiofrequency ablation of the occipital nerve into clinical question with some promise in early trials [19–21]. Neuromodulation via ONS provides a minimally invasive, adjustable, and reversible therapeutic option for those with refractory ON pain. Raoul et al. further demonstrated the utility of ONS on a cohort of 60 adult patients with refractory ON, which showed a 72.2% reduction in mean VAS score after one year of ONS device implantation [10].

Regardless of the etiology, ON pain is a disabling condition often leading to depression, loss of independence, and truancy in the pediatric population which potentially impacts long-term developmental success [4]. The treatment algorithm for pediatric patients with medically refractory ON pain mimics that for adults with ON and includes integration of preventative and abortive pharmacological strategies, occipital nerve blocks, and botulinum toxin injections among other interventions. Unfortunately, adult patients who continue to suffer are evaluated for neurosurgical management including ONS while pediatric patients are generally not [5, 7]. One obstacle is the general lack of the special skills required from subspecialized clinicians to elicit a pain characterization and history from a pediatric patient supportive enough to indicate that a child is a reasonable candidate for a trial of neuromodulation [2]. Additional challenges in pediatric patients include hardware-related issues such as implications for implanting devices designed for adults in children. These include the potential for growth-related hardware issues due to the accumulation of biomechanical strain and stress. There are additional ethical issues including the lack of FDA indications for the pediatric use of neuromodulation devices, informed consent/assent, and the impact of missing MRI safety labeling.

As in adults, open surgical strategies are implemented in some cases. Villeneuve et al. recently described significant improvement in the VAS (average 8.3 to 1) and statistically significant decrease in polypharmacy in a retrospective single-institutional series of six adolescent patients who underwent greater occipital nerve decompression or neurectomy for post-traumatic ON [22]. While effective, surgery caused sensory paresthesias in 100% of the patients (follow-up period was, on average, 10 months post-operatively). This ablative surgical approach therefore might be considered premature in the setting of an equally effective nondestructive strategy like ONS.

Although literature on ONS on the pediatric population is scant, and the pediatric data not discernible from the data from Borius and Valade and Vadivelu et al., it provides preliminary safety and efficacy data and substantiates our own single-institution findings [23, 24]. It is essential that further trials are undertaken to elucidate the long-term safety and efficacy of ONS as well as to examine for any adverse outcomes or device-related complications which may be unique to the pediatric population [10, 15, 23].

## Limitations

Durable conclusions about safety and efficacy of ONS in children are limited by our small sample size. The quality of data both in the literature and in our single institution study is, at best, Level III and lacks quality of life data granularity which would be improved by using standardized pain surveys.

## Conclusion

Medically refractory occipital neuralgia within the pediatric population is a source of severe disability and can be difficult to treat. ONS is an effective, non-permanent, and minimal-access intervention that has been shown to be effective for this condition. This study highlights the scarcity of available data on the indications and long-term effects of ONS within the pediatric population. Additional collaboration is necessary to improve outcomes, identify appropriate indications, and refine the surgical technique in children.

### Supplementary Information

Below is the link to the electronic supplementary material.

Supplementary file1 (JPG 130 KB)

## Data Availability

No datasets were generated or analysed during the current study.

## References

[1] Genizi J, Khourieh-Matar A, Assaf N, Chistyakov I, Srugo I (2017) Occipital headaches in children: are they a red flag? J Child Neurol 32(11):942–946. 10.1177/0883073817723266 28768455 10.1177/0883073817723266

[2] Eidlitz-Markus T, Zeharia A, Haimi-Cohen Y, Konen O (2014) Occipital and craniocervical pain and brain MRI in children with migraine. Pediatr Neurol 50(4):347–352. 10.1016/j.pediatrneurol.2013.11.004 24485928 10.1016/j.pediatrneurol.2013.11.004

[3] Irwin SL, Gelfand AA (2018) Occipital headaches and neuroimaging in children. Curr Pain Headache Rep 22(9):59. 10.1007/s11916-018-0712-6 29987497 10.1007/s11916-018-0712-6

[4] Kim S (2022) Pediatric headache: a narrative review. J Yeungnam Med Sci 39(4):278–284. 10.12701/jyms.2022.00528. Epub 2022 Sep 14. PMID: 36102115; PMCID: PMC9580058 10.12701/jyms.2022.00528 PMC9580058 36102115

[5] Barmherzig R, Kingston W (2019) Occipital neuralgia and cervicogenic headache: diagnosis and management. Curr Neurol Neurosci Rep 19(5):20. 10.1007/s11910-019-0937-8 30888540 10.1007/s11910-019-0937-8

[6] Love SM, Hopkins BD, Migdal CW, Schuster NM (2022) Occipital headache evaluation and rates of migraine assessment, diagnosis, and treatment in patients receiving greater occipital nerve blocks in an academic pain clinic. Pain Med 23(11):1851–1857. 10.1093/pm/pnac080 35595240 10.1093/pm/pnac080

[7] Ryu JH, Shim JH, Yeom JH, Shin WJ, Cho SY, Jeon WJ (2019) Ultrasound-guided greater occipital nerve block with botulinum toxin for patients with chronic headache in the occipital area: a randomized controlled trial. Korean J Anesthesiol 72(5):479–485. 10.4097/kja.19145 31159537 10.4097/kja.19145 PMC6781206

[8] Salmasi V, Olatoye OO, Terkawi AS, Hah JM, Ottestad E, Pingree M (2020) Peripheral nerve stimulation for occipital neuralgia. Pain Med 21(Supplement_1):S13–S17. 10.1093/pm/pnaa083 32804226 10.1093/pm/pnaa083

[9] Weiner RL, Reed KL (1999) Peripheral neurostimulation for control of intractable occipital neuralgia. Neuromodulation: Technology at the Neural Interface 2(3):217–221. 10.1046/j.1525-1403.1999.00217.x 22151211 10.1046/j.1525-1403.1999.00217.x

[10] Raoul S, Nguyen JM, Kuhn E et al (2020) Efficacy of occipital nerve stimulation to treat refractory occipital headaches: a single-institution study of 60 patients. Neuromodulation: Technology at the Neural Interface 23(6):789–795. 10.1111/ner.13223 32725745 10.1111/ner.13223

[11] Green A, Issa MA, Kim CH (2013) Peripheral nerve stimulation for treatment of chronic headache: a case report. W V Med J 109(6):24–28 24371861

[12] Johnson MD, Burchiel KJ (2004) Peripheral stimulation for treatment of trigeminal postherpetic neuralgia and trigeminal posttraumatic neuropathic pain: a pilot study. Neurosurgery 55(1):135. 10.1227/01.NEU.0000126874.08468.89 15214982 10.1227/01.NEU.0000126874.08468.89

[13] Keifer OP, Diaz A, Campbell M, Bezchlibnyk YB, Boulis NM (2017) Occipital nerve stimulation for the treatment of refractory occipital neuralgia: a case series. World Neurosurgery 105:599–604. 10.1016/j.wneu.2017.06.064 28634063 10.1016/j.wneu.2017.06.064

[14] Kapural L, Mekhail N, Hayek SM, Stanton-Hicks M, Malak O (2005) Occipital nerve electrical stimulation via the midline approach and subcutaneous surgical leads for treatment of severe occipital neuralgia: a pilot study. Anesth Analg 101(1):171. 10.1213/01.ANE.0000156207.73396.8E 15976227 10.1213/01.ANE.0000156207.73396.8E

[15] Slavin KV, Nersesyan H, Wess C (2006) Peripheral neurostimulation for treatment of intractable occipital neuralgia. Neurosurgery 58(1):112. 10.1227/01.NEU.0000192163.55428.62 16385335 10.1227/01.NEU.0000192163.55428.62

[16] Ghaemi K, Capelle HH, Kinfe TM, Krauss JK (2008) Occipital nerve stimulation for refractory occipital pain after occipitocervical fusion: expanding indications. Stereotact Funct Neurosurg 86(6):391–393. 10.1159/000175802 19033708 10.1159/000175802

[17] Liberati A, Altman DG, Tetzlaff J et al (2009) The PRISMA statement for reporting systematic reviews and meta-analyses of studies that evaluate healthcare interventions: explanation and elaboration. BMJ 339:b2700. 10.1136/bmj.b2700 19622552 10.1136/bmj.b2700 PMC2714672

[18] Staudt MD, Hayek SM, Rosenow JM et al (2023) Congress of Neurological Surgeons systematic review and evidence-based guidelines for occipital nerve stimulation for the treatment of patients with medically refractory occipital neuralgia: update. Neurosurgery 93(3):493. 10.1227/neu.0000000000002578 37458729 10.1227/neu.0000000000002578

[19] Pisapia JM, Bhowmick DA, Farber RE, Zager EL (2012) Salvage C2 ganglionectomy after C2 nerve root decompression provides similar pain relief as a single surgical procedure for intractable occipital neuralgia. World Neurosurg 77(2):362–369. 10.1016/j.wneu.2011.06.062 22120336 10.1016/j.wneu.2011.06.062

[20] Lozano AM, Vanderlinden G, Bachoo R, Rothbart P (1998) Microsurgical C-2 ganglionectomy for chronic intractable occipital pain. J Neurosurg 89(3):359–365. 10.3171/jns.1998.89.3.0359 9724107 10.3171/jns.1998.89.3.0359

[21] Abd-Elsayed A, Yapo SA, Cao NN, Keith MK, Fiala KJ (2023) Radiofrequency ablation of the occipital nerves for treatment of neuralgias and headache. Pain Pract. 10.1111/papr.13276 37461297 10.1111/papr.13276

[22] Villeneuve LM, Coulibaly NJ, Raza SM, Poinson B, Chrusciel D, Desai VR (2023) Surgical management of pediatric occipital neuralgia: a single-center experience of an uncommon pathology. J Neurosurg Pediatr 1(aop):1–8. 10.3171/2023.5.PEDS22554 10.3171/2023.5.PEDS22554 37548543

[23] Vadivelu S, Bolognese P, Milhorat TH, Mogilner AY (2012) Occipital nerve stimulation for refractory headache in the Chiari malformation population. Neurosurgery 70(6):1430. 10.1227/NEU.0b013e3182545a1c 22418582 10.1227/NEU.0b013e3182545a1c

[24] Borius PY, Valade D (2021) A new treatment option for children with refractory chronic paroxysmal hemicranias: occipital nerve stimulation. Pediatr Neurol 125:18–19. 10.1016/j.pediatrneurol.2021.09.010 34624605 10.1016/j.pediatrneurol.2021.09.010

