# Supplementary figures and images for: Occipital nerve stimulation in pediatric patients with refractory occipital neuralgia
Source: Childs Nerv Syst. 2024 Apr 2;40(8):2465–70. doi: 10.1007/s00381-024-06376-x (PMC11269409; doi:10.1007/s00381-024-06376-x)

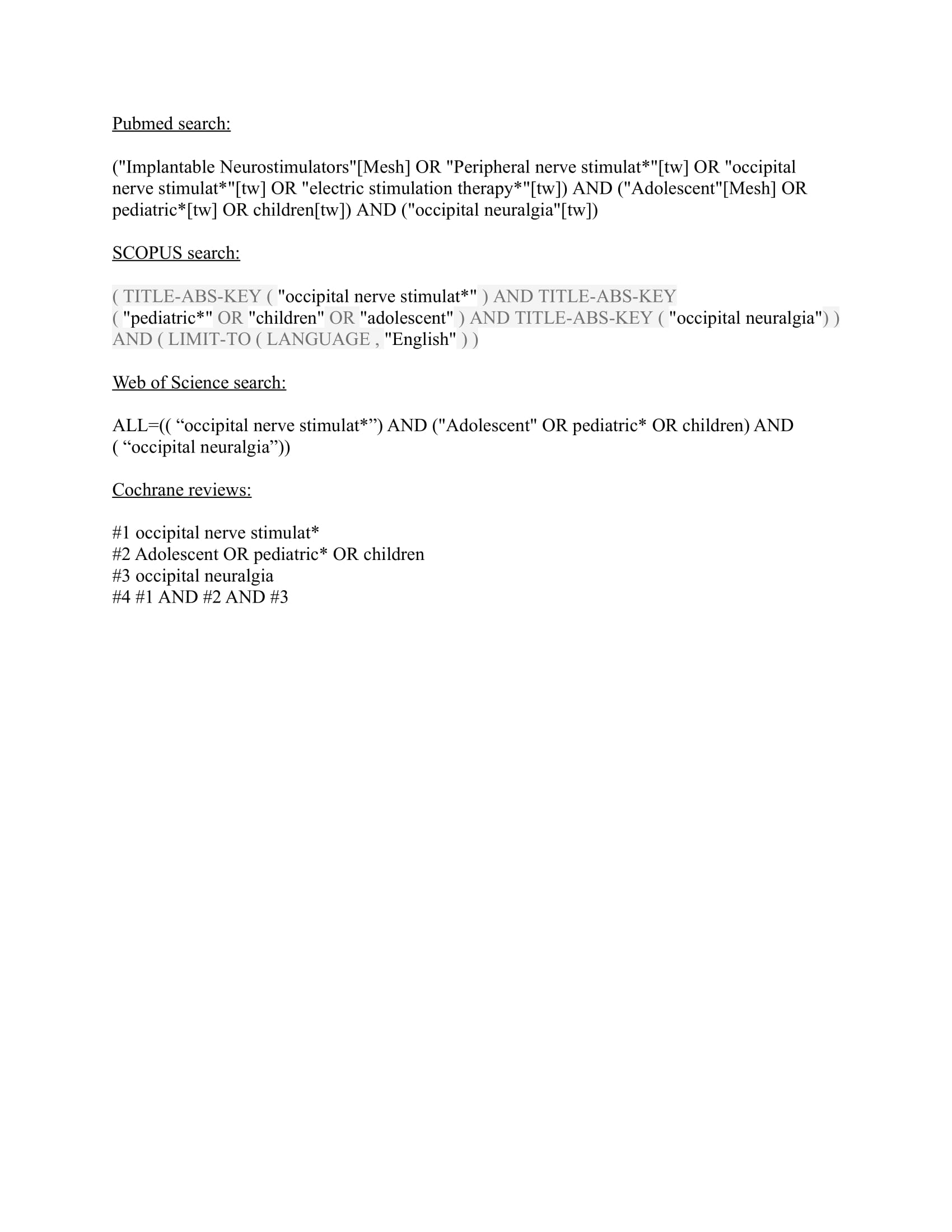

Supplement: Supplementary file 1 — Supplementary file1 (JPG 130 KB) [file 381_2024_6376_MOESM1_ESM.jpg]
